# HP1γ expression is elevated in prostate cancer and is superior to Gleason score as a predictor of biochemical recurrence after radical prostatectomy

**DOI:** 10.1186/1471-2407-13-148

**Published:** 2013-03-23

**Authors:** Jon Slezak, Matthew Truong, Wei Huang, David Jarrard

**Affiliations:** 1 School of Medicine and Public Health, University of Wisconsin-Madison, Madison, WI, 53750, USA; 2 Department of Pathology and Laboratory Medicine, University of Wisconsin-Madison, Madison, WI, 53750, USA; 3 Department of Urology, University of Wisconsin-Madison, Madison, WI, 53705, USA

**Keywords:** Prostate cancer, HP1γ, Prognosis, PSA-Recurrence, Ki-67, Tissue microarray

## Abstract

**Background:**

Aberrant chromatin structure in cancer cells results from altered proteins involved in its packaging. Heterochromatin protein 1 gamma (HP1γ) is a non-histone heterochromatic protein that functions to maintain chromatin stability and is important in embryonic development. Given an interest in the role developmental genes play in cancer, we investigated HP1γ expression in prostate cancer (PCa) and its prognostic associations.

**Methods:**

Tissue microarrays consisting of benign (N = 96), localized cancer (N = 146), metastatic PCa (N = 44), and HGPIN (N = 50) were immunoflourescently stained for HP1γ and Ki-67. Using a novel, automated quantitative imaging system, VECTRA™, epithelial staining in both the nucleus and cytoplasm was quantified and compared against clinicopathologic variables.

**Results:**

HP1γ is significantly elevated in HGPIN (80%), localized PCa (76%), and metastatic PCa (98%) compared to benign tissues from both the nuclear and cytoplasmic compartments (*P* < 0.0001). Increased nuclear and total HP1γ expression was associated with Gleason score (*P* = 0.02 and *P* = 0.04 respectively). Given known binding to the C-terminus of Ki-67, a co-expression analysis was performed that revealed a correlation between nuclear and cytoplasmic HP1γ and Ki-67 (Pearson Coefficient 0.321 and 0.562 respectively, *P* < 0.0001). Cox survival analysis demonstrated that cytoplasmic HP1γ expression was an independent prognostic marker and out-performed pathological Gleason score for predicting PSA-recurrence after radical prostatectomy.

**Conclusions:**

In this first detailed analysis of HP1γ expression in cancer, VECTRA™ demonstrates compartmentalized and total HP1γ protein expression is increased in PCa and that expression correlates with clinical outcomes better than Gleason score. Given the critical role HP1γ plays in chromatin organization and gene expression, it represents a novel prognostic and therapeutic target.

## Background

Prostate cancer (PCa) is the second most common cancer in men and will account for approximately 28,170 deaths in 2012 [1]. The behavior of some cancers can be variable despite Gleason score and other clinicopathologic factors. Recent efforts have focused on developing biomarkers that provide clinicians with the improved ability to identify clinically significant cancers and aid in treatment decisions. Genes important in embryogenesis frequently play a role in cancer [2]. One such gene, heterochromatin protein 1 gamma (HP1γ), is critically involved in chromatin packaging [3] and demonstrates altered expression during development and cell differentiation [4-6].

HP1γ, along with HP1α and HP1β, belong to a family of heterochromatin proteins with stabilizing functions. The structure of HP1 contains an evolutionarily conserved chromodomain that binds to a methylated lysine on histone H_3_ (H3K9me) [7]. This binding epigenetically marks regions of silenced or reduced gene expression [8]. HP1 stabilizes telomeric and centromeric heterochromatin structure and facilitates DNA repair after disruption or damage [9,10]. This chromatin repair function is important to maintain information encoded in the epigenetic histone code [9]. The HP1 isoforms appear to have differential functions. HP1γ has the ability to regulate both heterochromatin and euchromatin structure, while the other isoforms are localized only on heterochromatin.

Decreased expression of HP1γ occurs during cell differentiation which is unique to this isoform compared to HP1α and β [6]. In differentiated human tissues levels of HP1γ are generally not detectable [11]. Silencing of HP1γ has functional consequences. In selected cancer cell lines, growth is inhibited. In a recent screen of cancer tissues, HP1γ protein appeared to be overexpressed in samples of lung, breast, colon and esophageal [5]. Although decreased levels of its isoform HP1α were found in metastatic breast cancer compared to localized [12], HP1γ has not been previously evaluated as a marker for cancer progression.

Herein, we identify a novel overexpression of HP1γ in PCa tissues using a tissue microarray (TMA) and VECTRA™ image acquisition technology. This quantitative tool permits localization of expression to nuclear versus cytoplasmic compartments. We report that nuclear and cytoplasmic HP1γ is progressively increased in HGPIN, PCa, and metastatic PCa cores compared to benign tissue. Furthermore, increased HP1γ is correlated with Ki-67, a protein known to complex with other heterochromatin family proteins [13]. Finally, we report a novel prognostic role for cytoplasmic HP1γ in independently predicting PSA recurrence following prostatectomy. The use of high-throughput imaging technologies such as VECTRA allows for quantitative compartmentalization of marker expression to predict cancer outcomes and lends further insight into tumor biology.

## Methods

### Tissue microarray

The University of Wisconsin Institutional Review Board (IRB) provides ethical insight to clinical projects and reviews all human research protocols in accordance with federal regulations, state laws, and local and University policies. FFPE-patient tissues were obtained from the University of Wisconsin Department of Pathology and Laboratory Medicine under IRB approval. A tissue microarray was constructed using tissues from 64 PCa patients (mean age, 62.8 years) collected from 1995 to 2006. The mean follow-up period for this cohort was 9.6 years. The TMA consists of 336 duplicate cores from different disease groups: 43 localized PCa (pT2), 30 aggressive PCa (pT3 and 4), 21 metastatic PCa, 23 high grade intraepithelial neoplasia (HGPIN) (tissue from HGPIN tissue blocks of some of the PCa patients of this cohort) and 48 benign prostate tissues (BPT) (from the non-tumor blocks of some of the cancer patients in this cohort).

### Staining

Slide preparation and antigen retrieval were conducted as previously described [14]. Briefly, the TMA slides were taken through routine deparaffinization and rehydration, pretreated with endogenous peroxidase block and retrieval buffer. Slides were then rinsed with dH2O, then Tris Buffered Saline (TBS), then TBS with Tween (TBST), followed by protein blocking at room temperature. E-cadherin antibodies (Cell Signaling Technology, Beverly, MA) were used to define the epithelial compartment for better tissue segmentation. Slides were then stained with antibodies against HP1γ (EMD Millipore, Bilerica, MA) [15] and Ki-67 (Abcam, Cambridge, MA).

### Image analysis

For automated image acquisition and analysis, the stained slides were loaded onto the slide scanner. Slides were scanned as previously described [14]. Cores with <5% epithelial component or loss of tissue were excluded from the analysis. Nuance system and inForm 1.2™ software (Caliper Life Sciences, Hopkinton, MA) were used to for building spectral libraries on a per-cell basis for HP1γ and Ki-67 target signals according to manufacturer’s protocols. This system allows automated quantitation of fluorescent staining on a per-cell basis and selection of cellular subsets (nucleus versus cytoplasmic) for analysis of target signals.

### Statistical analysis

Nuclear and cytoplasmic expression of individual cores of various prostate tissues (benign, HGPIN, PCa, metastatic PCa) was statistically compared using the t-test. Correlation analysis was used to assess the relationship between HP1γ and Ki-67 expression in each compartment. We then assessed the relationship between HP1γ expression and patient clinicopathologic features using t-tests and analysis of variance (ANOVA). For this analysis, multiple PCa cores obtained from the same patient were first averaged, providing a more precise estimation of core expression in each biological replicate. HP1γ expression was then compared with clinicopathologic information (PSA, Gleason, pT stage, tumor volume, SV involvement, margins, extracapsular extension, and evidence of metastasis) collected from the 64 patients included in this study. Univariate and multivariate Cox regression analyses were used to assess whether nuclear and cytoplasmic HP1γ (as continuous variables) predicted biochemical recurrence following prostatectomy. Statistically significant variables from univariate Cox analysis were then entered into a multivariate Cox regression model. Variables that were not independent predictors of recurrence were removed using backward selection (*P* < 0.05). SPSS Version 20.0 (IBM, Armonk, New York) was used to perform statistical analyses. All tests were two-tailed and a *P* value < 0.05 was considered statistically significant.

## Results

### HP1γ expression in prostate core samples is elevated in PCa, HGPIN, and metastatic PCa

**Figure 1:**
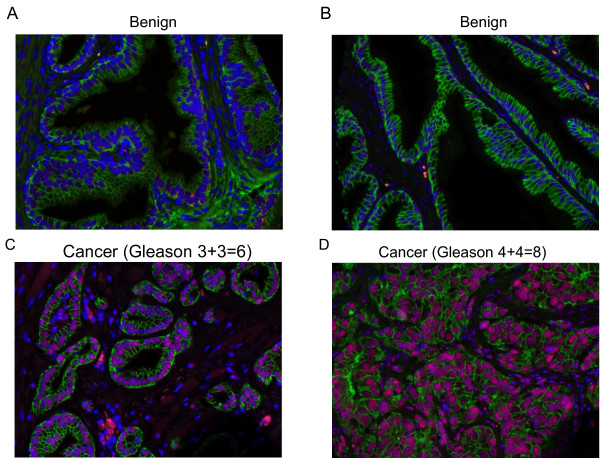
**HP1γ expression in human prostate cancer is increased compared to benign normal prostate tissue.** Tissue microarrays (336 cores) containing benign (**A** and **B**), HGPIN, low grade (**C**), high grade (**D**) and metastatic PCa were immunoflourescently stained for HP1γ (red). E-cadherin (green) labels epithelial cells and DAPI (blue) the cell nuclei. Increased staining is noted in PCa samples with nuclear expression greater than cytoplasmic. Low levels of HP1γ expression were infrequently observed in benign epithelial cells. In contrast, HP1γ was uniformly upregulated in the majority of prostate cancer cells.

**Figure 2:**
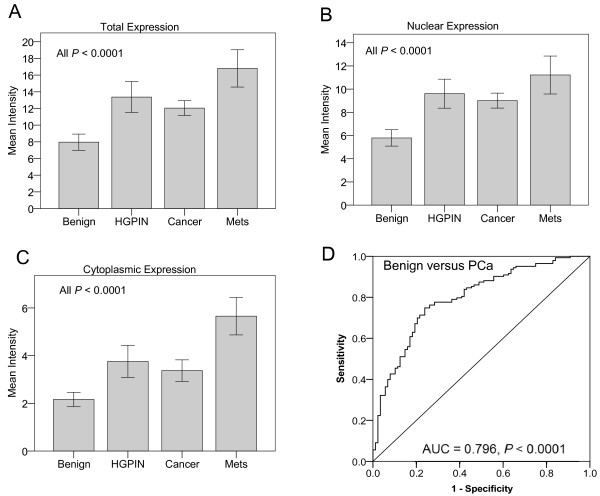
**Quantitative analysis of HP1γ expression in human prostate cancer.** VECTRA™ technology was used for automated image attainment and analysis per the manufacturer’s protocol. Both the nuclear and cytoplasmic compartments were compared. Cell intensities were averaged on a per cell basis for each core to determine the mean intensity of nuclear, cytoplasmic and total HP1y expression. Mean intensity of expression of HP1y was then compared for benign, HGPIN, cancer and metastasis core samples with 95% confidence intervals. HP1y expression in HGPIN, cancer (N= 146 cores), and metastasis is significantly greater than benign (N=96 cores) for total (**A**), nuclear (**B**), and cytoplasmic (**C**) expression with *P* values <0.0001. Nuclear expression of the protein was ~2.5 fold greater than cytoplasmic expression. **D**) ROC curve analysis was used to determine the optimum cut-off for defining overexpression. Total HP1γ expression demonstrated excellent discrimination between benign and cancer (AUC = 0.796, *P* < 0.0001). A cut-off of 9.095 maximized the sensitivity (76.2) and specificity (73.5%) and was used to define overexpression.

We measured HP1γ expression on TMAs using VECTRA™ quantitative immunofluorescent analysis. Expression was measured in both the nuclear and cytoplasmic compartments of epithelial and stromal cells. In normal prostate tissues, HP1γ was expressed at low levels. Total expression of HP1γ was significantly increased in HGPIN and PCa, and was highest in metastatic tumors compared to benign samples (Figure 1). In the epithelial compartment, total, nuclear, and cytoplasmic expression were increased in PCa specimens compared to benign (Figure 2A-C) (*P*-values <0.0001). Nuclear levels of HP1γ were 2–3 fold higher than cytoplasmic staining for all benign and cancer samples.

To define overexpression, ROC curve analysis was performed using total HP1γ expression to identify the optimum cut point that maximized the sum of the sensitivity and specificity for discriminating between benign versus cancer cores. ROC analysis demonstrated excellent discrimination between benign versus cancer (AUC = 0.796, *P* < 0.0001) with a sensitivity and specificity of 76.2% and 73.5%, respectively (Figure 2D). Using the optimum cut-off of 9.095 to define overexpression, 109/143 (76.2%) of localized PCa cores, 40/50 (80%) of HGPIN cores, and 40/41 (97.6%) of metastatic PCa cores demonstrated overexpression of total HP1γ compared to only 23/88 (26.1%) of benign cores.

#### Ki-67 expression correlates with increased HP1γ expression in PCa

**Figure 3:**
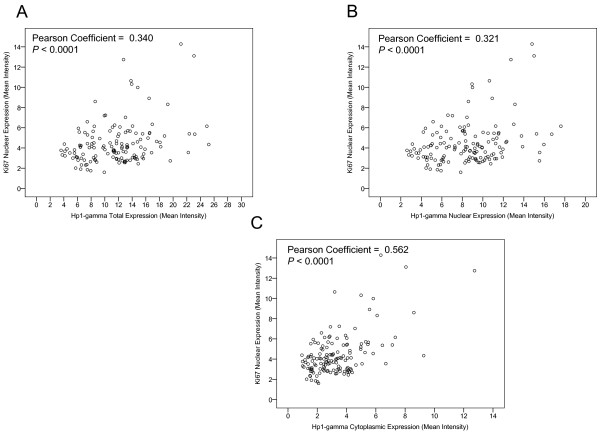
**HP1γ expression correlates with Ki-67.** VECTRA™ technology was used for automated image attainment and analysis per the manufacturer’s protocol to assess immunoflourescent staining of Ki-67 and HP1γ. Pearson correlation coefficients yielded significant relationships between mean intensity expression of nuclear Ki-67 and **A**) total ( *P* = 0.34), **B**) nuclear ( *P* = 0.32), and **C**) cytoplasmic ( *P* = 0.562) HP1γ. All *P* values <0.0001.

Ki-67 is a known marker of cell proliferation that has been demonstrated to complex with HP1γ on heterochromatin [13,16,17]. We assessed the correlation between HP1γ and Ki-67 on a per core basis. Within nuclear and cytoplasmic compartments there was a significant correlation between nuclear Ki-67 and HP1γ expression (Figure 3A-C). The strength of the correlation was stronger for cytoplasmic than nuclear expression (Pearson Coefficients 0.562 and 0.321, respectively, *P* <0.0001).

#### HP1γ expression is associated with Gleason score and pT category

A series of clinicopathologic patient variables including seminal vesicle involvement, tumor volume, Gleason score, margins, the presence of metastases, and extracapsular extension were compared to HP1γ nuclear and cytoplasmic expression. Using HP1γ expression as a continuous variable, we did not find an association of the majority of these variables with HP1γ expression (Table 1). However, the analysis did find that Gleason score was associated with nuclear and total expression (*P* = 0.018 and *P* = 0.042, respectively). In summary, HP1γ expression does not appear to be tightly associated with many standard clinicopathologic variables.

**Table 1:** Association of Hp1γ expression with patient pathological features

***Variable***	***Number***	**Nucleus**		**Cytoplasm**		**Total**	
		**Mean intensity (SD)**	**p-value**	**Mean intensity (SD)**	**p-value**	**Mean intensity (SD)**	**p-value**
Gleason							
3+3 or 3+4	47	8.77 (2.5)	0.018	3.02 (0.8)	0.095	11.8 (3.2)	0.042
4+3 or 4+4 or 4+5	12	10.74 (2.5)		4.1 (2.0)		14.9 (4.5)	
Stage							
T2	40	8.62 (2.7)	0.238	2.86 (0.9)	0.039	11.48 (3.4)	0.12
T3	12	9.46 (1.6)		3.47 (0.7)		12.92 (2.2)	
T4	11	10.0 (2.9)		3.77 (2.0)		13.78 (4.7)	
Tumor Volume							
<5	7	9.59 (1.8)	0.776	3.07 (0.8)	0.156	12.67 (2.4)	0.879
5-20	36	8.84 (2.9)		3.06 (1.4)		11.91 (4.0)	
>20	20	9.09 (2.4)		4.01 (2.5)		12.03 (3.1)	
SV involvement							
No	49	8.96 (2.6)	0.684	3.04 (1.2)	0.182	12.01 (3.7)	0.468
Yes	11	9.30 (2.5)		4.76 (3.0)		12.29 (3.3)	
Margins Positive							
No	41	9.40 (2.6)	0.105	3.22 (1.3)	0.469	12.55 (3.7)	0.876
Yes	21	8.25 (2.5)		3.0 (1.0)		11.25 (3.4)	
Extracapsular							
No	46	8.91 (2.8)	0.633	3.39 (2.0)	0.875	11.83 (3.8)	0.471
Yes	17	9.26 (2.0)		3.31 (0.9)		12.57 (2.8)	
Metastasis							
No	52	8.95 (2.7)	0.723	3.11 (1.2)	0.640	12.07 (3.7)	0.682
Yes	12	9.25 (2.04)		4.50 (3.1)		11.97 (2.7)	

#### HP1γ expression predicts PSA recurrence

**Figure 4:**
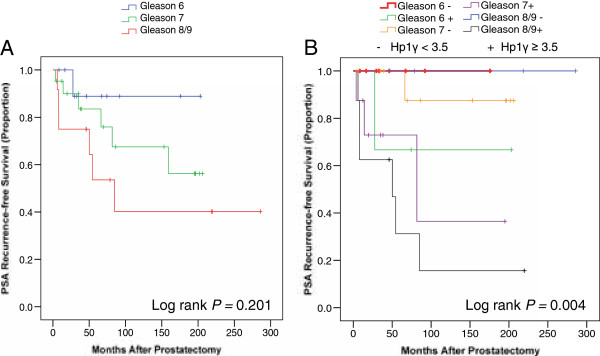
**Kaplan Meier analysis comparing pathological Gleason score versus Hp1γ.** PSA failure after radical prostatectomy was examined in samples stained for Hp1γ. **A**) Gleason score provides minimal separation of patients into risk categories for biochemical recurrence using a Log rank analysis (*P* = 0.2). **B**) Cytoplasmic Hp1γ is superior to pathological Gleason score when Gleason score was stratified into high versus low Hp1γ. The median value for Hp1γ staining in was 3.5 in all benign and cancer samples and this value was subsequently used as a cut point to define high versus low cytoplasmic Hp1γ expression for the Log rank analysis. Hp1γ accurately predict biochemical recurrence within each Gleason category including intermediate grade cancer (GS 6, 7).

Univariate Cox survival analysis demonstrated that Gleason score, stage (pT), seminal vesicle involvement, metastasis, and cytoplasmic HP1γ expression predicted biochemical recurrence (*P* < 0.05) (Table 2). Both nuclear and cytoplasmic HPγ expression levels were analyzed as continuous variables. Furthermore, using multivariate Cox regression, we demonstrated that only the presence of metastasis and cytoplasmic HPγ were independent predictors of biochemical recurrence following prostatectomy (*P* = 0.014 and *P* = 0.038, respectively) (Table 3). Gleason score was no longer a statistically significant predictor of biochemical recurrence on multivariate analysis (*P* > 0.05). To verify that cytoplasmic Hp1γ was a strong predictor of recurrence than Gleason score, we performed a Kaplan Meier analysis of Gleason score alone (Figure 4A) versus each Gleason score stratified by high versus low cytoplasmic HP1γ expression (Figure 4B). Use of HP1γ allowed excellent separation of Gleason 7 patients into high (65%) versus low (15%) probability of biochemical recurrence. Furthermore, high HP1γ expression correctly predicted recurrence in 10/11 (91%) of patients. However, with the Gleason system, having high-grade disease (Gleason 8 or 9) was only able to correctly identify 6/12 (50%) cases of recurrence.

**Table 2:** Univariate cox regression analysis for predicting biochemical recurrence

Variables*	**Coefficent**	**s.e.**	**Wald**	***P***	**Odd ratio (95% CI)**
Gleason category	0.392	0.197	4.025	0.045	1.485 (1.009-2.186)
Stage (pT) category	0.860	0.355	5.853	0.016	2.363 (1.177-4.741)
Tumor volume	0.051	0.298	0.029	0.864	1.052 (0.587-1.887)
Seminal vesicle involvement	1.195	0.542	4.860	0.027	3.305 (1.142-9.566)
Margins	−0.154	0.574	0.072	0.789	0.858 (0.278-2.641)
Extracapsular extension	0.792	0.513	2.386	0.122	2.208 (0.808-6.033)
Metastasis	1.414	0.516	7.489	0.006	4.112 (1.495-11.312)
Nuclear Hp1γ (continuous)	0.043	0.107	0.165	0.685	1.044 (0.847-1.287)
Cytoplasmic Hp1γ (continuous)	0.751	0.356	4.449	0.035	2.118 (1.055-4.255)

**Table 3:** Multivariate cox regression analysis for predicting biochemical recurrence

Variables*	**Coefficent**	**s.e.**	**Wald**	***P***	**Odd ratio (95% CI)**
*Remained in model: (P < 0.05):*					
Metastasis	1.690	0.690	6.005	0.014	5.418 (1.402-20.930)
Cytoplasmic Hp1γ (continuous)	0.899	0.432	4.320	0.038	2.456 (1.053-5.732)

*Variables removed from the model (*P* > 0.05): Gleason category, stage (pT) category, seminal vesicle involvement.

## Discussion

Patients and clinicians are in need of more accurate biomarkers to predict the prognosis of prostate cancer, especially for intermediate grade tumors [18]. Few markers have been reported that reliably predict treatment failure (e.g. PSA recurrence after surgery). Altered nuclear shape and size are a hallmark of cancer and reflect changes in chromatin structure. Image analyses of heterochromatin content and nuclear shape have been reported to improve the prediction of PCa prognoses [19]. HP1γ is a member of the HPI family of proteins involved in chromatin packaging and gene regulation. The current study represents the first in depth analysis of HP1γ expression for any cancer. We observe an increase in HP1γ expression in the majority of prostate cancers. Furthermore, by analyzing staining in distinct cellular compartments in a cohort of patients with extended follow-up, we find a role for the expression of this gene in predicting treatment failure.

A unique strength of this study lies in our use of the newly validated VECTRA™ imaging technology. The platform merges automated slide scanning, multi-spectral imaging technology, and pattern recognition software into a system for biomarker analysis. VECTRA™ has many advantages over other imaging systems for biomarker quantitation [14]. VECTRA™ allows researchers to objectively analyze expression within separate epithelial and stromal tissue compartments. Nuclear and cytoplasmic staining within these compartments is concurrently assessed and multiple markers may be addressed simultaneously. The system is highly automated and uses pattern recognition to measure expression on a per-cell, core, or compartment basis on TMAs. The algorithm designed to segment tissue compartments by our genitourinary pathologist [WH] had a 97% rate of acceptable tissue segmentation. In the current analysis, VECTRA™ permitted a reproducible assessment of HP1γ, and its association with Ki-67, in multiple cell compartments for a large cohort of patients.

HP1γ and the other HP1 family members have been primarily studied during embryogenesis and development, including that of the prostate [4]. Expression of HP1γ was noted in the fetal prostate at 14 and 24 weeks of gestation. This is in contrast to HP1α which is not expressed at those developmental stages [4]. Decreased expression of HP1γ is required for normal cellular differentiation [5,6]. Our study demonstrates that expression of HP1γ occurs in 98% of metastatic and 76% of localized PCa tumors compared to 26% of benign tissues. This cut-off was determined by ROC analysis to highlight the marked differences between patients with benign versus cancer. Adjusting this cut point for Hp1-gamma overexpression can increase the sensitivity or specificity depending on its required use. Total expression was associated with higher Gleason score (p = 0.04).

One notable finding was that HP1γ was not only an independent predictor of PSA-recurrence for patients who underwent radical prostatectomy, but was superior to pathological Gleason score using both a multivariate Cox model and a Kaplan-Meier analysis (Table 3 and Figure 4). Few biomarkers to date are able to predict PSA recurrence more robustly than Gleason score. Recently, Cuzick *et al.* reported a panel of 31 RNA markers had a more significant *P* value compared to Gleason score for predicting PSA recurrence [20]. One explanation for the robustness of HP1γ is that its prognostic value is not collinearly associated with other clinicopathologic variables (Table 1). Given the role HP1γ plays in gene repression [3,21] and development [4], we postulate that aberrant expression of this protein may be central to the pathophysiology of PCa.

Increased expression of HP1γ in the nucleus compared to the cytoplasm is consistent with data demonstrating this protein is primarily localized to centromeric and telomeric heterochromatin [3,8] and euchromatin [22,23]. It was of interest to find expression of the protein in the cytoplasm and note that this carried prognostic significance. Its presence in the cytoplasm may represent altered protein processing, or may have some as of yet unknown functional significance. Ki-67 is a nuclear protein that correlates with cell proliferation and is a known marker of PCa progression [24]. The C-terminal domain has been found to bind to all three members of the HP1 family [13]. We found a significant association between HP1γ and Ki-67 on a per core basis (Figure 2). Given the more uniform expression of HP1γ across prostate cancer cells when compared to Ki-67, it clearly has other functions independent from Ki-67 in the regulation of heterochromatin.

## Conclusion

In conclusion, we demonstrate that HP1γ expression is elevated in PCa and this independently predicts PSA-recurrence for patients more accurately than pathological Gleason score. Use of novel biomarkers to identify men at lower risk for recurrence may reduce the need for unnecessary adjuvant radiation therapy. HP1γ represents a novel marker of prostate cancer progression that will be useful to the clinician with further validation in other datasets. In addition, examination of HP1γ expression in biopsy specimens might fulfill an urgent need for more accurate pre-treatment risk stratification tools. Given the role HP1γ plays in epigenetic gene regulation through its binding to methylated lysine on histone H_3_ (H3K9me) [7], it represents a novel target for cancer treatment. Furthermore, a recent survey of tumors suggests HP1γ may be overexpressed in other epithelial cancers [3].

## Abbreviations

PCa: Prostate cancer; HP1γ: Heterochromatin protein γ; H3K9me: Histone 3 lysine 9 methylated; TMA: Tissue microarray; GS: Gleason score.

## Competing interests

The authors declare that they have no competing interests.

## Authors’ contributions

JS, MT, and DJ analyzed the results and wrote the paper. MT performed statistical analysis. WH carried out the experiments and operated VECTRA™ technology. DJ designed the study and provided supervision. All authors read and approved the final manuscript.

## Pre-publication history

The pre-publication history for this paper can be accessed here:

http://www.biomedcentral.com/1471-2407/13/148/prepub
